# Variation in the susceptibility of urban *Aedes* mosquitoes infected with a densovirus

**DOI:** 10.1038/s41598-020-75765-4

**Published:** 2020-10-29

**Authors:** Aurélie Perrin, Anne-Sophie Gosselin-Grenet, Marie Rossignol, Carole Ginibre, Bethsabée Scheid, Christophe Lagneau, Fabrice Chandre, Thierry Baldet, Mylène Ogliastro, Jérémy Bouyer

**Affiliations:** 1 UMR MIVEGEC, CNRS, IRD, Univ Montpellier, Montpellier, France; 2 UMR DGIMI, INRAE, Univ Montpellier, Montpellier, France; 3 EID-med, Entente Interdépartementale pour la Démoustication du littoral méditerranéen, Montpellier, France; 4 ASTRE, Cirad, INRAE, Univ Montpellier, Montpellier, France; 5 Insect Pest Control Sub-Programme, Joint Food and Agriculture Organization/International Atomic Energy Agency, Programme of Nuclear Techniques in Food and Agriculture, 1400 Vienna, Austria

**Keywords:** Invasive species, Virology, Virus-host interactions

## Abstract

Urban *Aedes* mosquitoes are vectors of many viruses affecting human health such as dengue, chikungunya and Zika viruses. Insecticide resistance and environmental toxicity risks hamper the effectiveness of chemical control against these mosquito vectors. Alternative control methods, such as the use of mosquito-specific entomopathogenic viruses should be explored. Numerous studies have focused on evaluating the potential of different densoviruses species as biological control agents. However, knowledge on the extent of inter- and intra-specific variations in the susceptibility of *Aedes* mosquitoes to infection by different densoviruses remains insufficient. In this study, we compared infection and mortality rates induced by the *Aedes albopictus* densovirus 2 in different strains of *Aedes albopictus* and *Aedes aegypti* mosquitoes. The two *Aedes* species were different in terms of susceptibility to viral infection. Under laboratory conditions, Aedes albopictus densovirus 2 appeared more virulent for the different strains of *Aedes aegypti* tested than for those of *Aedes albopictus*. In addition, we also found significant intra-specific variation in infection and mortality rates. Thus, although even if Aedes albopictus densoviruses could be powerful biocontrol agents used in the management of urban *Aedes* populations, our results also call into question the use of single viral isolate as biocontrol agents.

## Introduction

*Aedes albopictus* (the tiger mosquito) and *Ae. aegypti* (the yellow fever mosquito), are particularly invasive species that proliferate in tropical and temperate urban environments and are the main vectors of dengue, chikungunya, yellow fever and more recently Zika viruses. In the context of globalization and the movement of goods and people, these emerging vector-borne diseases are now present on almost every continent^1,2^.

In the absence of vaccine or antiviral therapy for the majority of these diseases, vector control is the main strategy to prevent their spread. This is mainly practiced by controlling adult mosquito populations through spatial treatments, in emergency situation, using pyrethroid-based chemical insecticides and by controlling larvae through physical suppression of breeding sites or larvicides. The application of insecticides can be problematic because of their high environmental and human health toxicity^3–6^, their general toxicity to non-target insects^7^, and the insecticide resistance of target mosquitoes^8,9^. Pyrethroids are the most widely used chemical insecticides in the world but their intensive use has led to the selection of pyrethroid resistant mosquitoes worldwide^8,10,11^. Many innovative approaches are being developed to control *Aedes* sp*.* mosquitoes such as adult traps, lethal ovitraps, autodissemination stations, release of insects with dominant lethality (ridl), sterile insect technique, incompatible insect technique^12^ but larval control remains essential and is systematically included in any integrated control strategy.

The control of urban *Aedes* larvae is extremely complex to implement because of the diversity and multitude of larval habitats, which are made up of small, and usually cryptic, water containers^13^. Apart from chemical larvicides (e.g. temephos, pyriproxyfen, diflubenzuron), the biological larvicide recommended against urban *Aedes* larvae is derived from *Bacillus thuringiensis* subsp*. israelensis* (*Bti*), a natural soil bacteria selected for its exclusive pathogenic action on some species of Diptera^14^. However, its effectiveness is limited by many biological and environmental factors: sunlight, amount of organic matter, larval density and depth of breeding sites^15^. For the moment, no resistance to *Bti* has been observed in mosquito but it is crucial to develop other candidate larvicides to fulfil the range of effective and environmental-friendly control tools. The use of several bio-larvicides with different action spectra should ensure effective, feasible and sustainable vector control and should contribute to manage the resistance of target insects to the active molecules.

Many viruses are known to be pathogenic for mosquitoes^16–18^, but their potential use in biological control has been limited by their low infectivity or a production method unsuitable for field treatment. Mosquito densoviruses (MDVs), that exhibit a narrow host range and multiple transmission patterns, are, however, a potential alternative^19,20^. Densoviruses (DVs), also known as densonucleosis viruses, are small icosahedral non-enveloped DNA viruses belonging to the *Parvoviridae* family and are highly infectious for invertebrate (insects, crustaceans and echinoderm)^21,22^. MDVs have been isolated from laboratory colonies or natural populations of mosquitoes and from chronically infected mosquitoes-derived cell lines. Within the *Brevidensovirus* genus, there are currently two type species with 9 virus species (i) the *Dipteran brevidensovirus 1* with Aedes aegypti densovirus 1 and 2 (AaeDV1^23^ and AaeDV2^24^ respectively), Aedes albopictus densovirus 1 (AalDV1^25^), Culex pipiens pallens densovirus (CppDV^26^) and Anopheles gambiae densovirus (AgDV^27^), (ii) the *Dipteran brevidensovirus 2* with Aedes albopictus densovirus 2 and 3 (AalDV2^28^ and AalDV3^29^ respectively) and Haemagogus equinus densovirus (HeDV^30^). In the genus *Ambidensovirus,* only one strain has been described, the Culex pipiens densovirus (CpDV^31^). Five others viruses are described in literature but are not yet included in the official taxonomy. Three strains of Aedes albopictus densoviruses, AalDV4^32^ to AalDV6 have been isolated and described from C6/36 cell line without any cytopathic effect suggesting that persistent cryptic infections are common^30^. The sequences of these viruses are sufficiently different that it is highly unlikely that they have evolved from a single contamination event^30^. Despite the lack of cytopathic effect in cell lines, theses MDVs have been shown to be pathogenic to mosquito larvae by oral infection and are able to replicate and to be transmitted in adult mosquitoes. Unlike strains isolated from cell lines, a new strain, AalDV7, was isolated from field-collected *Ae. albopictus*^33^. The Aedes Thai strain densovirus (AthDV) was detected in colonies of *Ae. aegypti* and *Ae. albopictus* from Thailand^34^. MDVs can infect a wide range of mosquitoes, but natural infection appears to be confined to a single host species. The host range has been more or less well described according to the viruses. AaeDV1, the best characterized of them, was infectious in laboratory experiments for *Ae. aegypti, Ae. albopictus, Ae. cantans, Ae. caspius, Ae. geniculatus, Ae. vexans, Cx. pipiens* and *Culiseta annulata*^19^*.* MDVs are thought to persist in nature by horizontal transmission from larvae to larvae in the wild aquatic environments, although transovarial and sexual transmission have also been recorded^3,20,27,33–37^. They are highly pathogenic for larvae at all stages, but mortality is higher when infection occurs at an early stage. Older larvae can survive and grow into imago after the virus infection, and infected adult female mosquitoes can transmit the virus vertically to the next generation^38^. Mortality is also higher during critical phases of mosquito life, especially during larval metamorphosis, pupation and adult emergence, which require more energy.

MDVs are emerging as promising tools for the control of *Aedes* mosquito population. The objective of the ERC Revolinc project, that funded this study, is to use these biopesticides to boost the sterile insect technique^39^. Sterile males would thus be coated with these viruses before being release, thus contaminating wild females even in the absence of successful mating^39^. Most studies have been devoted to evaluating the potential of different MDVs strains as biocontrol agents. Knowledge on the extent of inter- and intra-specific variations in the susceptibility of *Aedes* mosquitoes to DVs infection are lacking. The overall objective of this study was to determine the potential of AalDV2 as a biological control agent against *Aedes* urban mosquitoes. AalDV2 (formerly known as AaPV for Aedes albopictus parvovirus) was isolated from a chronically infected cell line of the C6/36 clone of *Ae. albopictus*^28,40,41^ and was found to be highly pathogenic for *Ae. aegypti* neonate larvae reaching 95% mortality in the N’ Goye strain. No data are available on the pathogenicity of this virus to *Ae. albopictus*. In this work, we assessed the pathogenicity of AalDV2 against different strains of *Ae. albopictus* and *Ae. aegypti* mosquitoes from different geographical areas.

## Results

### Susceptibility of different strains of urban *Aedes* to AalDV2 infection

**Figure 1:**
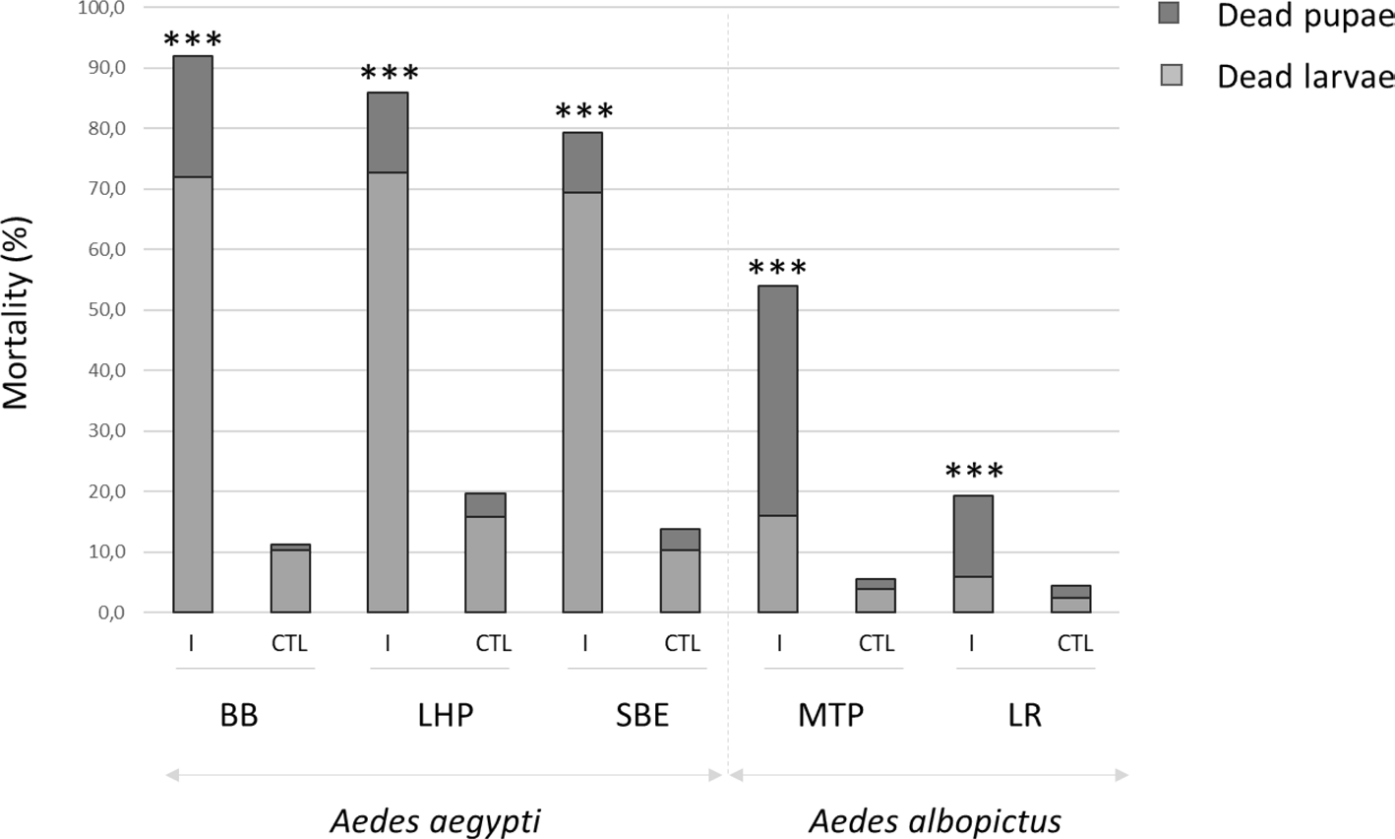
Percentage of cumulative mortality, corresponding to dead larvae or pupae, 25 days following AalDV2 infection of first instar mosquito larvae from different strains of *Ae. albopictus* (MTP, Montpellier and LR, La Réunion) and *Ae. aegypti* (LHP, Long-Hoà, BB, Bora-Bora and SBE, Benin). Larvae (N = 3 replicates of 150 larvae per experimental condition) were infected, or not, with 3 × 10^11^ veg/ml of AalDV2. Binomial linear mixed effect models were used to compared the impact of AalDV2 in infected groups (I) compared to control groups (CTL). ***p-value < 0.001.

Three replicates of 150 first-instar larvae of each strain of *Ae. albopictus* and *Ae. aegypti* were, or not, exposed to AalDV2. Larval mortality appeared 5–7 days after infection for *Ae. aegypti* strains and 6–9 days after infection for *Ae. albopictus* strains. For all strains, peak of mortality occurred before adult emergence, between 6 and 10 days post infection (p.i.). Figure 1 shows the percentage of cumulative mortality on day 25 after infection, corresponding to dead larvae or pupae as well as individuals that disappeared uring the experiment, in the control (CTL) and infected (I) groups. In the control groups, the mortality rate was about 5% for *Ae. albopictus* strains and up to 20% for *Ae. aegypti* Long-Hoa Permethrin strain (LHP) at the end of the experiment. Infection with AalDV2 caused mortality in both species. However, under the same rearing and bioassay conditions, *Ae. aegypti* strains showed a higher cumulative mortality rate than *Ae. albopictus* strains (Table S1, p < 0.001) suggesting that AalDV2 is more pathogenic for this species.

Differences in intra-specific susceptibility to the virus were also observed. In *Ae. aegypti*, the cumulative mortality of larvae and pupae infected with AalDV2 was up to 92% for the Bora Bora strain (BB), 86% for the LHP strain and 79% for the Benin strain (SBE). Mortality rates of controls without infection were similar (Table S2, p = 0.247) for BB and SBE strains, and higher than those observed for LHP (p < 0.001). Viral infection increased mortality of all strains (p < 0.001), but more for BB than for LHP and SBE (p < 0.001). In *Ae. albopictus* strains, we observed 55% mortality in AalDV2-infected mosquitoes for the Montpellier strain (MTP) and 19% for the La Réunion strain (LR). The impact of the virus on mortality was significant (Table S3, p < 0.001) and greater for the MTP strain than for the LR strain (p < 0.001). Mortality occurred at different stages of mosquito development. Larval mortality in *Ae. aegypti* was 73% for the LHP, 72% for the BB and 69% for the SBE strains; compared to 16% for the MTP and 6% for the LR strains of *Ae. albopictus.* Pupal mortality in *Ae. albopictus* was 38% for the MTP and 13% for the LR strains; compared to 13% for the LHP, 10% for the SBE and 20% for the BB strains of *Ae. aegypti*. Overall, there were significant differences in the pathogenicity of AalDV2 between the different strains of *Aedes* mosquitoes. Mortality was lower and occurred later for the tested strains of *Ae. albopictus* than for the *Ae. aegypti* strains.

**Figure 2:**
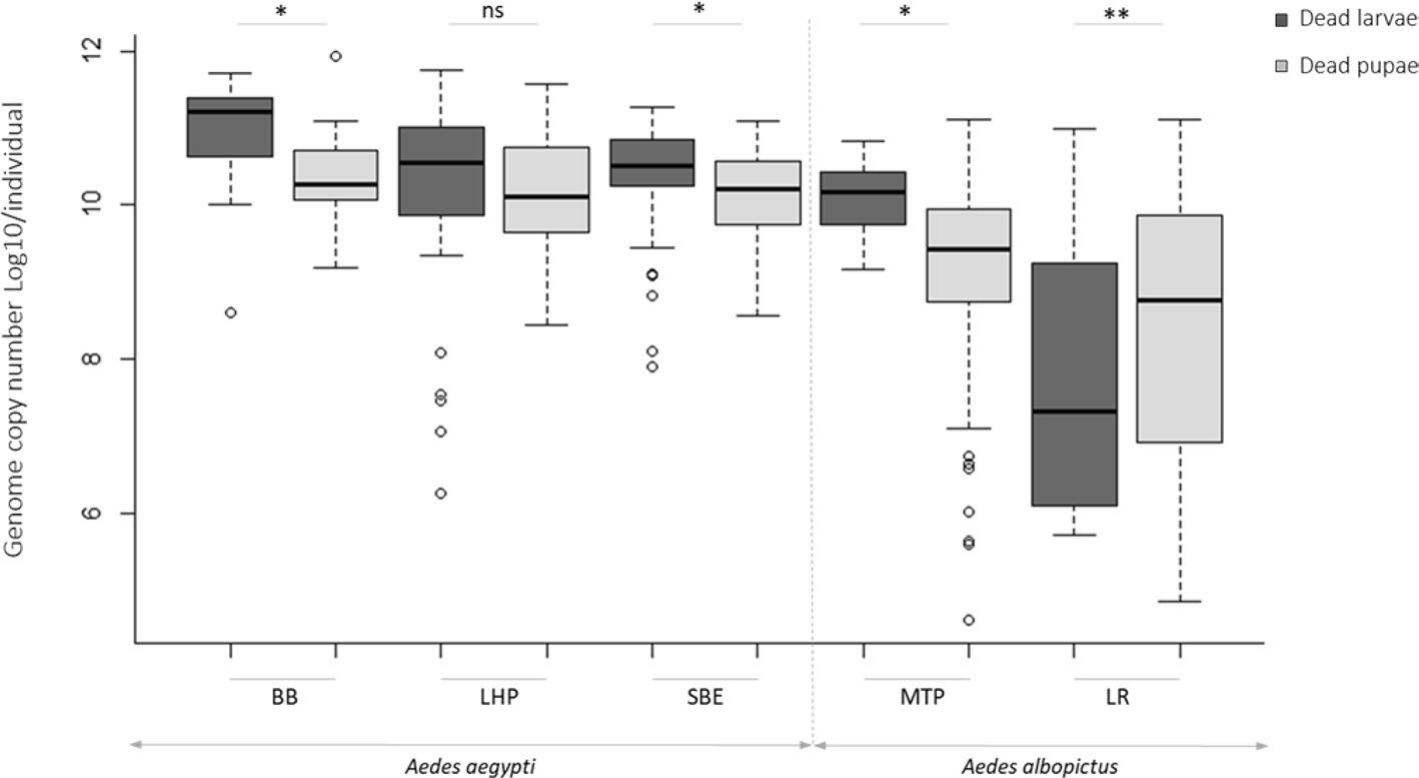
Viral dose in dead larvae or pupae of AalDV2-infected mosquitoes for each tested strain of *Ae. aegypti* (LHP, Long-Hoà, BB, Bora-Bora and SBE, Benin) and *Ae. albopictus* (MTP, Montpellier and LR, La Réunion). Larvae (N = 3 replicates of 150 larvae per experimental condition) were infected, or not, with 3 × 10^11^ veg/ml of AalDV2. Viral doses of dead larvae and dead pupae are expressed as log 10 of the number of AalDV2 genomes quantified (gev) by qPCR in each individuals. Wilcoxon W test was used to compared virus titer in larvae compared to pupae for each strain. ***p-value < 0.001; *p-value < 0.05; n.s., not statistically significant.

Dead mosquitoes were collected daily and analysed by qPCR for detection and quantification. In the infected groups, the virus was detected in all dead mosquitoes both in the larval and pupal stages (100%, N = 1322). Control mosquito were negative for any densoviruses infection. Moreover, no virus detection was observed in the control mosquito samples that died during the experiment (N = 234). Figure 2 shows the viral dose quantified in dead larvae and pupae for each strain tested of *Ae. albopictus* and *Ae. aegypti*. Viral doses were slightly higher in larvae than pupae, except for the LR strain of *Ae. albopictus* where pupae were significantly more infected than larvae (Wilcoxon test, W = 645, p < 0.0001).

Table 1 shows the mean viral copy number of the AalDV2 for each strain tested according to stage of development. In *Ae. aegypti* larvae, viral titers reached 5.14E + 11 gev/individual in BB strain, 1.49E + 11 gev/individual in LHP strain and 1.33E + 11 gev/individual in SBE strains with an average of 1.73E + 11 ± 6.72E + 10 gev/individual for BB strain, 8.89E + 10 ± 4.51E + 10 gev/individual in LHP strain and 4.40E + 10 ± 8.51E + 09 gev/individual in SBE strain. In larvae of *Ae. albopictus*, viral titers were less important and reached 6.81E + 10 gev/individual in MTP strain and 1.12E + 09 gev/individual in LR strain with an average of 1.85E + 10 ± 5.7E + 09 gev/individual in MTP strain and 1.20E + 08 ± 1.98E + 08 gev/individual in LR strain. In pupae, viral titer was on average significantly lower than in larvae, although we observed very high titer in some individuals, including in *Ae. albopictus*. Indeed, the viral titers reached 8.71E + 11 gev/individual in the BB strain, 3.80E + 11 gev/individual in the LHP strain, 1.26E + 11 gev/individual in the SBE strain, 1.27E + 11 gev/individual in the strain MTP and 1.30E + 11 gev/individual in the LR strain. On average, we obtain 7.60E + 10 ± 8.76E + 10 gev in the BB strain, 5.03E + 10 ± 4.15E + 10/individual in the LHP strain, 2.44E + 10 ± 9.98E + 09 gev/individual in the SBE strain of *Ae. aegypti*. In pupae of *Ae. albopictus*, viral titers were less important excepted for LR strain. We obtained an average of 9.83E + 09 ± 4.6E + 09 gev/individual in MTP strain and 1.35E + 10 ± 9.86E + 09 gev/individual in LR strain.

**Table 1:** Mean viral copy number of AalDV2 in dead larvae and pupae for each tested strain of *Ae. aegypti* (LHP, Long-Hoà, BB, Bora-Bora and SBE, Benin) and *Ae. albopictus* (MTP, Montpellier and LR, La Réunion).

	Virus titer in dead larvae (gev/individual)	Virus titer in dead pupae (gev/individual)
BB^a^	1.73E + 11 ± 6.72E + 10	7.60E + 10 ± 8.76E + 10
LHP^a^	8.89E + 10 ± 4.51E + 10	5.03E + 10 ± 4.15E + 10
SBE^a^	4.40E + 10 ± 8.51E + 09	2.44E + 10 ± 9.98E + 09
MTP^b^	1.85E + 10 ± 5.87E + 09	9.83E + 09 ± 4.60E + 09
LR^b^	1.20E + 08 ± 1.98E + 08	1.35E + 10 ± 9.86E + 09

Larvae (N = 3 replicates of 150 larvae per experimental condition) were infected or not, with 3 × 10^11^ veg/ml of AalDV2. Viral doses of dead larvae and pupae are expressed as the number of AalDV2 genomes quantified by qPCR in each individuals (gev/individual).

^a^*Aedes aegypti*; ^b^*Aedes albopictus.*

### Impact of densovirus infection on the predation of infected larvae

**Figure 3:**
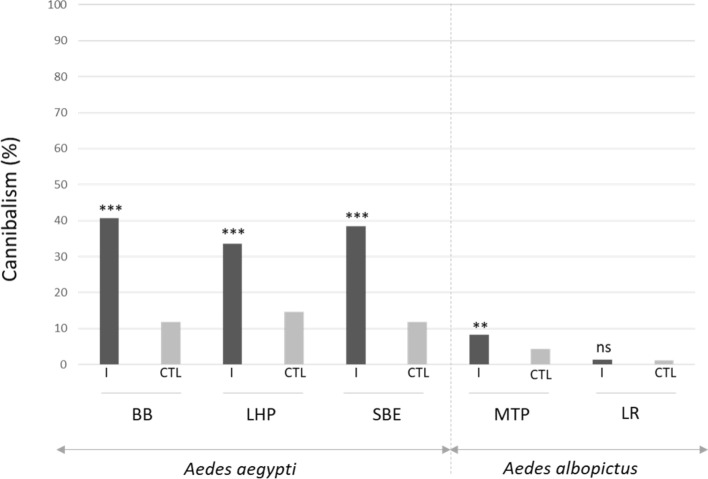
Larval cannibalism rate on day 25 after AalDV2 infection of first instar mosquito larvae for the different strains tested of *Ae. albopictus* (MTP, Montpellier and LR, La Réunion) and *Ae*. *aegypti* (LHP, Long-Hoà, BB, Bora-Bora and SBE, Benin). Larvae (N = 3 replicates of 150 larvae per experimental condition) were infected or not, with 3 × 10^11^ veg/ml of AalDV2. Binomial linear mixed effect models were used to compared the impact of AalDV2 on cannibalism in infected groups (I) compared control groups (CTL). ***p-value < 0.001; **p-value < 0.01; n.s., not statistically significant.

At the end of the experiment, all individuals were counted. Missing individuals between the start (n = 150/replicate) and the end of the trial were considered as consumed by the others. Under these conditions of infection and rearing, *Ae. albopictus* strains are significantly less prone to predation after DVs infection than *Ae. aegypti* strains (Table S4, p < 0.001). As shown in Fig. 3, in the *Ae. albopictus* strains, we observed little losses with only 8.2% in the MTP strain (Table S6, p < 0.05) and 1.3% in the LR strain (p = 0.399). We frequently observed high losses in infected groups of *Ae. aegypti*. Missing larvae in AalDV2-infected mosquitoes reached 40.7% in the BB strain, 33.6% in the LHP strain and 38.5% in the SBE strain. Viral infection increased the loss of larvae in all strains of *Ae. aegypti* (Table S5, p < 0.001), but more so in the BB and SBE strains than in the LHP strain (p < 0.001).

### Densovirus infection on surviving adults

**Figure 4:**
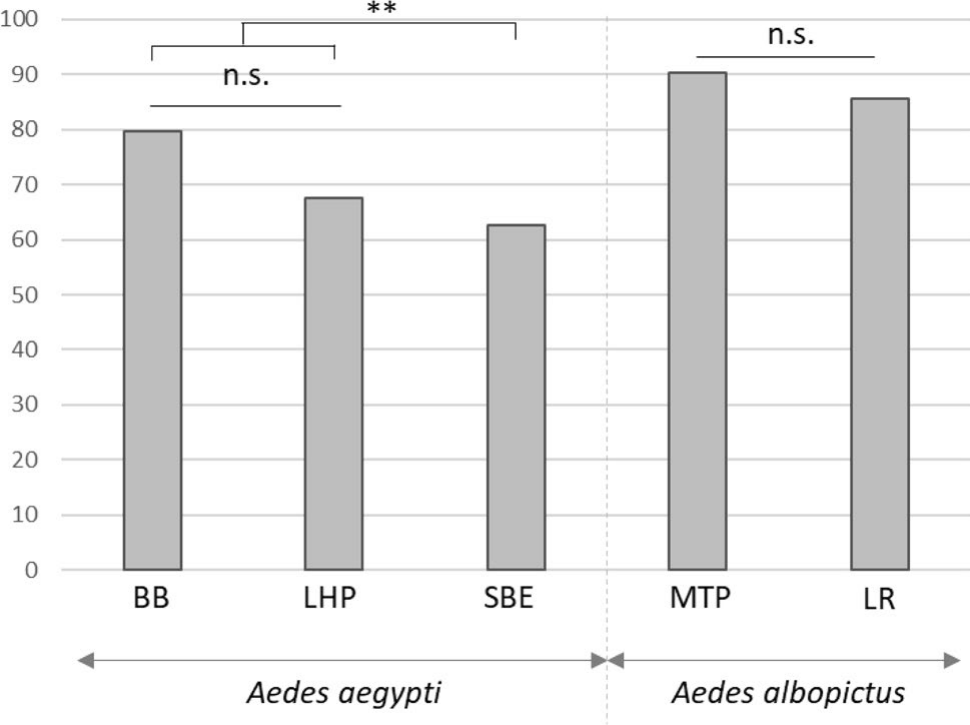
Percentage of infected adults surviving after AalDV2 infection of first instar mosquito larvae for the different strains tested of *Ae. albopictus* (MTP, Montpellier and LR, La Réunion) and *Ae*. *aegypti* (LHP, Long-Hoà, BB, Bora-Bora and SBE, Benin). Larvae (N = 3 replicates of 150 larvae per experimental condition) were infected or not, with 3 × 10^11^ veg/ml of AalDV2. Binomial linear mixed effect models were used to compared the impact of AalDV2 in different strains. **p-value < 0.01; n.s., not statistically significant.

Surviving emerged adults (males and females) were collected daily and analyzed by qPCR for AalDV2 detection and quantification. The infection rate in surviving adults was very high: the virus was still present after the emergence of adults at a significant level. As shown in Fig. 4 the prevalence of AalDV2 in surviving adults was higher in *Ae. albopictus* strains than in *Ae. aegypti* strains, with comparable prevalence rates between MPT (90.3%) and LR (85.7%). In *Ae. aegypti*, surviving adults of the SBE strain are significantly less infected than the two other strains (Table S7, p < 0.01). Thus, we observed that 62.6% (72 of 115) of surviving adults of the SBE strain, 67.6% (23 of 34) of the LHP strain, and 79.6% (43 of 54) of the BB strain of surviving adults were infected with AalDV2. The overall frequency of infection did not vary by gender. In *Ae. albopictus*, 87.9% of females and 92.7% of males for the MTP strain were infected with AalDV2 compared to 82% of females and 92.7% of males for the LR strain. In *Ae. aegypti,* the prevalence rates of AalDV2 infection were 60% of females and 78.6% of males for the LHP strain, 66.2% of females and 56.1% of males for the SBE strain, and 74.4% of females and 93.3% of males for the BB strain.

The viral dose in surviving infected adults was not significantly different between the different strains of *Ae. albopictus* and *Ae. aegypti*. In *Ae. aegypti*, viral titers reached 3.61E + 10 gev/individual for the BB strain, 1.84E + 10 gev/individual for the LHP strain and 3.19E + 10 gev/individual for the SBE strains with an average of 3.45E + 09 ± 2.04E + 09 gev/individual for the BB strain, 2.23E + 09 ± 2.17E + 09 gev/individual for the LHP strain and 2.02E + 09 ± 1.30E + 09 gev/individual for the SBE strain. Similarly, in *Ae. albopictus*, viral titers reached 3.42E + 10 gev/individual for the MTP strain and 3.41E + 10 gev/individual for the LR strain with an average of 3.12E + 09 ± 1.37E + 09 gev/individual for the MTP strain and 2.92E + 09 ± 1.21E + 09 gev/individual for the LR strain. The viral dose was not significantly different in surviving adults by gender.

The surviving emerged adults were sorted by sex. Table 2 shows the sex ratio between males and females by infection status and species. Imbalances in sex ratios were observed in the infected groups compared to control groups for SBE (p < 0.05) and BB (p < 0.001) strains of *Ae. aegypti* but not for LHP strain (p = 0.1). No differences were observed in *Ae. albopictus* MTP (p = 0.81) and LR (p = 0.83) strains.

**Table 2:** Sex ratio (female number/male number) in control and infected groups for each strains of *Ae. aegypti* (LHP, SBE, BB) and *Ae. albopictus* (MTP, LR) (*p < 0.05; ***p < 0.001; Chi-squared test).

	Female	Male	sex-ratio F/M	χ^2^, df = 1, p value
**LHP**^a^				
Control	117	141	0.83	2.65, p = 0.10
Infected	21	14	1.5	
**SBE**^a^				
Control	135	134	1.01	5.30, *p < 0.05
Infected	73	43	1.70	
**BB**^a^				
Control	111	138	0.80	13.98, ***p < 0.001
Infected	38	14	2.71	
**MTP**^b^				
Control	111	115	0.97	0.05, p = 0.81
Infected	32	31	1.03	
**LR**^b^				
Control	154	144	1.07	0.04, p = 0.83
Infected	128	124	1.03	

^a^*Aedes aegypti*; ^b^*Aedes albopictus.*

## Discussion

Originally established from homogenates of mosquito larvae^42^, *Ae. albopictus* C6/36 cells are highly permissive to many arboviruses^43^ and are widely used for screening mosquito field collections. Aedes albopictus Densovirus 2, AalDV2, was first described in a C6/36 cell sub-line during a study on arboviruses in African mosquitoes^28^. Its origin is unknown but is probably due to contamination by samples of infected mosquito collected in the field. Our results showed that not only *Ae. aegypti* but also, for the first time, *Ae. albopictus*, were both susceptible to oral infection with AalDV2. However, we showed intra-specific and inter-specific variation in infection between the five different strains of mosquitoes tested, including insecticide resistant strains. The Long-Hoà (LHP, Vietnam) strain of *Ae. aegypti* and the La Réunion (LR) strain of *Ae. albopictus* are both pyrethroid-resistant strains. We have shown that these two strains are as susceptible to viral infection as the other strains. Most notably, AalDV2 appeared to be more pathogenic for *Ae. aegypti* than for *Ae. albopictus*. Mortality was mainly observed at larval stage in *Ae. aegypti* strains, compared to *Ae. albopictus* strains where less than 20% of larvae died before pupation. Viral titers of dead individuals were higher in larvae than in pupae and in *Ae. aegypti* strains than in *Ae. albopictus* strains. This suggests that clearance of the virus could occur between each larvae moult and be released into the rearing water during the moulting process. The virus appeared to replicate less in *Ae. albopictus* strains, which could explain its lower impact on this species. AalDV2 was isolated from persistently infected cell lines derived from *Ae. albopictus* which may have reduced the virulence of the densovirus strain to this species. Studies using C6/36 densoviruses on *Ae. albopictus* are poorly documented. A single study on AalDV1 has shown that this isolate seems to be very pathogenic for the Guangdong *Ae*. *albopictus* strain (China)^38^. However, the mortality observed since one day post-infection is questionable and may not be linked to the virus, but to environmental factors or to rearing conditions.

We also observed a variation in susceptibility to infection at the intra-specific level. Indeed, for *Ae. aegypti*, the Bora Bora strain (BB) had a statistically higher mortality rate after exposure to AalDV2 compared to the two others strains, Long-Hoà (LHP) and Benin (SBE). This difference in intra-specific susceptibility could be a consequence of the lower genetic variability of the BB strain associated with its older establishment in the insectarium compared to the two others strains. Similarly, for *Ae. albopictus*, the Montpellier strain (MTP) had a much higher mortality than the La Réunion strain (LR). The dynamics of infectious diseases can be affected by genetic diversity within host populations^44^ as well as by the time of colonization of laboratory populations of *Aedes* mosquitoes^45^. Previous studies using MDVs have shown a high level of mortality for the same species of mosquito exposed to different isolates of DVs, with some isolates being highly pathogenic, others more benign. For example, as they are different viral strains, AalDV1 infection resulted in a mortality rate of over 90% in 1st instar *A. aegypti* larvae^28^, while AaeDV infection results in a mortality of 75.1% for the same species^46^. Thus, after infection with AthDV, first-instar larvae of the Thai strain of *Ae. albopictus*, had a mortality of 82% compared to 51% for *Ae. aegypti*^34^. Different DVs strains such as AaeDV1, AthDV, AalDV2 and AalDV3 induced completely different levels of mortality when infecting Rexville D, Chachoengsao, and Bangkok strains of *Ae. aegypti* after 48 h exposure to DVs at 2 × 10^10^ gev/larvae^47^. Recently, analysis of sublethal effects also showed that AalDV7 infection of *Ae. aegypti* and *Ae. albopictus* first instar larvae significantly decreased pupation and emergence rates^33^. However, the diversity of experimental designs used does not allow easy comparison of the results across studies, due to differences in infection methods, environmental conditions, viral titers, and stage of larvae infected. Some intrinsic factors such as their genetic background, or their microbiota, could influence the susceptibility of mosquitoes to DVs infection but these mechanisms need to be explored. For example, in *Bombyx mori* lepidoptera, resistance to Bombyx mori densovirus type 1 or 2 (BmDV1 & BmDV2) is controlled by recessive non-susceptibility genes, nsd-1 and -2^48,49^, which affect distinct stages of the viral infection pathway^50^. In addition, a recent study has shown that *Wolbachia pipientis* infection promotes the replication of the Aedes albopictus densovirus 1 (AalDV1) in *Aedes* cell lines in a density dependent manner^51^. Further studies are needed to determine how these laboratory results may result in increased susceptibility to DVs in natural populations of *Wolbachia*-infected *Ae. albopictus* or artificially *Wolbachia*-infected *Ae aegypti*. Furthermore, the pathogenicity, prevalence and infection rate of MDVs may also vary with on environmental factors such as temperature, or other conditions such as larval density, method of infection and duration of exposure to MDVs^40,52^. The environmental factors that influence the efficacy of MDVs have not been thoroughly studied and further research is needed before these MDVs can be eligible for operational use in mosquito control programs.

In mosquitoes, cannibalism between larval instars of the same species has been frequently observed, especially in the later instars, with food deficiency or excessively high larval densities applied under rearing conditions^53^. Cannibalism is also an effective route of transmission for some pathogens, including DVs, when healthy larvae consume moribund infected ones. Sick larvae become lethargic in the later stages of infection and are unable to defend themselves against aggressive conspecifics^54^. Under our laboratory conditions, *Ae. albopictus* and *Ae. aegypti* had a larval development cycle of 9–10 days at 26 °C from egg-laying to emergence. During our experiment, infected larvae of *Ae. aegypti* showed a delay in development compared to unexposed larvae and especially compared to infected larvae of *Ae. albopictus*. After infection in the first-instar stage, larval development of infected *Ae. aegypti* larvae was heterogeneous compared to healthy larvae which have a more synchronized life cycle. Most of the cadavers collected were third and fourth instar larvae, while first or second instar larvae were rarely found. As larval mortality was checked and dead larvae were removed daily, we hypothesise that dead or dying larvae (infected with densovirus) were consumed by uninfected or less infected larvae through cannibalism (i.e. active killing and consumption of conspecifics) or necrophagy (i.e. consumption of dead conspecifics). The high incidence of larval predation observed in *Ae. aegypti* mosquitoes, a species more susceptible to AalDV2, suggests that this is an important pathway for the transmission and pathogenicity of this MDVs.

Adult mortality has not been evaluated in this work, but many studies have shown that DVs infection of mosquito larvae affects the life traits of infected adults^33,34,37^. Thus, the effects of sublethal infection of *Ae. aegypti* larvae by different isolates of DVs included extended larval development times, reduced pupal and adults weight, decreased fertility of females, and decreased adult lifespan. Although the ability to modify adult life characteristics, in particular fertility, differs between MDVs strains and mosquito species, a reduction in reproductive success could potentially lead to a decrease in mosquito density and vector capacity.

Females infected in the larval stage can transmit DVs vertically by laying infected eggs in new oviposition sites, resulting in the spread of MDVs in the mosquito population and an increased coverage and efficacy^55^. A semi-field trial have shown that adult female *Ae. aegypti* oviposition behavior led to successful AaeDV dispersal from treated breeding sites to new breeding sites in large-scale cages. However, the AaeDV titers achieved in the contaminated sites were not sufficient to reduce larval densities^36^. Further research is needed to assess for other MDVs whether this vertical transmission translates into operational efficiency in the field, which would be possible after one or more amplification cycles, as suggested by Carlson^19^. Analysis of infection rates and titers in live adults revealed that virus replication occurred in all strains for both *Aedes* species tested. Most surviving adults were positive to AalDV2 detection after infection of first instar larvae. Infection rates in surviving adult of *Ae. albopictus* was higher than that of *Ae. aegypti* reaching 90%. Virus replication occurred in all strains for both mosquito species tested with an average viral titre of 6.5–11 log/after potential exposure of first larval stage to 10 log of virus. Among the few emerging adults, the viral titer of AalDV2 infected females and males did not vary significantly. However, in some mosquito’s strains, we observed a distortion of the sex-ratio in favour of females. Butchasky et al. also observed a mortality rate in adult males infected at the larval stage with DVs three times higher than that females^56^. This selection could be of interest for spreading and maintaining the virus by vertical transmission from female to the offspring.

The results of this laboratory study provide baseline data on the susceptibility of *Ae. albopictus* and *Ae. aegypti* to AalDV2 infection. We have shown that the different strains of mosquitoes were susceptible to AalDV2 infection including for insecticides resistance strains. These results confirm even more the advantage of the isolate AalDV2 and of the DVs in general as a biological control agent. The differences in the pathogenicity of AalDV2 among *Aedes* mosquito strains draw attention to the risk associated with the development of single viral strain for use as biocontrol agents. Our work also suggest that DVs strains isolated from heterologous mosquito species may be more efficient against a given species, probably because they are less well adapted. In addition, highly pathogenic DVs that kill larvae before they reach adulthood may exert high selective pressure, which would increase the risk of resistance and decrease their efficacy over time. Thus, AalDV2 could be a potential mosquito control agent, but further research and development work is still needed, including studies on the different routes of transmission and its persistence in the environment through semi-field evaluations. The continued discovery and isolation of new MDVs will enrich the pool entomopathogenic mosquito viruses and provide a variety of choices for one or more combinations of MDVs to optimally target *Aedes* mosquitoes. The ability to produce the virus on a large-scale at low cost and in sufficient quantities is also required before innovative formulations can be developed for operational use with other methods as part of integrated vector management. The development of innovative formulations suitable for (i) direct use in breeding site or (ii) dissemination by the insects themselves according to the entomovectoring principle developed by the boosted SIT approach, is underway^39^.

The main limitation of this study is the number of strains used. We focused our study on the infectivity of a mosquito densovirus, AalDV2, on five different strains of *Aedes albopictus* and *Ae. aegypti*. Other densoviruses strains and other mosquito strains could be used to support our results.

## Methods

### Virus and cells line

We used two *Ae. albopictus* derived C6/36 cell lines^42^. The first, a chronically infected sub-line, was used as the source of the Aedes albopictus densovirus 2 (AalDV2)^28^. A second, free of any DVs, was used as a control. Both sub-lines were grown at 28 °C in RPMI medium (Dutcher, France), supplemented with 10% heat-inactived fetal calf serum (Gibco, USA), 1% non-essential amino-acids (Gibco, USA) and 1% penicillin–streptomycin (Gibco, USA). Cells were seeded at 7-days intervals in 25 cm^2^ flasks at 1:5 dilution. Infected cells were scraped into supernatant and kept at − 20 °C as virus stock. The viral concentration was estimated as described below. The titer, expressed as genome equivalent virus (gev), remained stable at 3 × 10^11^ gev/ml.

### Mosquito strains

Three different colonized strains of *Ae. aegypti* and two of *Ae. albopictus* have been used to study the pathogenicity of AalDV2 (Table 3).

**Table 3:** *Aedes albopictus* and *Ae. aegypti* mosquitoes strains.

Species	Strains	Origin	Lab colonization	Status
*Ae. aegypti*	BB	French Polynesia	1980	Susceptible^a^
	SBE	Benin	2008	Susceptible^a^
	LHP	Vietnam	1997	Resistant^b^ (*kdr*)
*Ae. albopictus*	MTP	France	2016	Susceptible^a^
	LR	Overseas France	2016	Resistant^b,c^

*BB* Bora Bora, *SBE* Bénin, *LHP* Long-Hoà, *MTP* Montpellier, *LR* La Réunion.

^a^Susceptible to all insecticides class.

^b^Resistant to pyrethroids, the main insecticide class used for *Aedes* vector control, *kdr:* knockdown resistance.

^c^The characterization of molecular and metabolic mechanisms of insecticide resistance is underway.

The reference strain of *Ae. aegypti* BB (Bora-Bora, French polynesia) and SBE (Benin) have been colonized for many years and were devoid of any phenotypic resistance to World Health Organization (WHO) susceptibility tests at diagnostic doses for the most common chemical insecticides or any known mechanism of insecticide resistance. The LHP (Long-Hoà, Vietnam), pyrethroid-resistant strain of *Ae. aegypti*, is homozygous for the knockdown resistance (*kdr*) gene^57^. In addition, we used two recently colonized *Ae. albopictus* strains from France. The La Réunion strain (LR), is a pyrethroid-resistant strain colonized since 2010 but whose resistance mechanism is currently under investigation. The Montpellier strain (MTP) susceptible to all insecticides has been colonized since 2016. Adult colonies are maintained in the Vectopole insectarium of IRD in Montpellier, France, at 28 °C, 70% humidity with a 14 h/10 h light/dark cycle and fed with 10% sugar solution. Female adult mosquitoes were artificially fed through a Parafilm-membrane (Hemotek membrane feeding systems, UK) using fresh rabbit blood kindly provided by IRD animal facilities. The larvae were reared at 28 °C in 2 l jars and fed with alevin powder. At 28 °C, pupae were obtained 6–7 days after immersion of the eggs in water and imago 2 days later.

### Virus infection of mosquito larvae from *Ae. aegypti* and *Ae. albopictus*

Newly hatched first instar *Aedes* larvae were infected as follow. Mosquito eggs were allowed to hatch in tap water with a 7.5% solution of 50:50 alevin powder and rabbit pellets. Twenty-four hours after hatching, pools of 150 larvae were exposed to 3 × 10^11^ gev/ml of cells infected with AalDV2, theoretically corresponding to 10^10^ gev per larvae, in a total volume of 5 ml and kept without food for 48 h. The control groups, were exposed to healthy C6/36 cells under identical conditions to those of the treatment groups. Two days after infection, larvae were transferred to 300 ml water bowls with food and observed daily until pupation. Pupae were transferred to new small cups of clear water and allowed to emerge into mosquito cages. Dead larvae or pupae, were collected daily and stored at − 20 °C for further investigations. The emerged adults were collected daily and sorted by sex. The larval, pupal and cumulative mortality was evaluated at the end of bioassays at day 25 after infection. The adult mortality was not assessed. Three biological replicates of each strain were performed.

The cumulative mortality observed at the end of the experiment takes into account the number of dead larvae and pupae, plus larvae lost during the experiment due to predation.

### Virus detection and quantification in mosquitoes

Quantification of the virus by qPCR was performed using the LightCycler 480 System (Roche, France) and specific primers designed in the non-structural gene NS1 (q*Aal*DV2-F: 5′-TggCCAACAATTACgAACAA-3′ and q*Aal*DV2-R: 5′-CTCTggAgCCgCTgTgTAAT-3′). A standard curve (10^9^ to 10^3^ viral genome copies per reaction) was generated using tenfold serial dilutions of p*Aal*DV2, a plasmid encompassing the entire *Aal*DV2 sequence^58^. The reactions were carried out in a 10 μl reaction mixture containing 5 µl of 1 × SyberGreen master mix I, 1.7 µl of DNase/RNase-free water, 0.4 µM of each primers and 2.5 µl of sample. Each sample was processed in triplicate under the following conditions: 95 °C for 3′, 45 cycles of 94 °C for 10 s, 60 °C for 10 s and 72 °C for 10 s. The data were analyzed using Light Cycler 480 software (Roche, France). Virus concentration in mosquitoes was determined individually and titers were expressed as genome equivalent virus (gev) per individual. Each individual (larvae, pupae or adult) was crushed in 50 µl of 0.1 × Tris–EDTA buffer supplemented with 0.05 µM salmon sperm DNA and the suspension was clarified for 5 min at 5000*g* before quantification.

### Statistical analysis

Binomial linear mixed effect models were used to analyse the impact of AalDV2 infection on survival at intra- and inter-specific level, cannibalism and infection rates in surviving adults (response variables). The mosquito species and strain as well as the infection status were used as fixed effects and the repetitions as random effects. Fixed-effects coefficients of all models and their corresponding p-values are reported in Tables S1 to S7. Data from viral concentration in dead or surviving individuals were analysed with Wilcoxon W test. Data from sex-ratio bias were analysed with chi-square test. By convention, results were considered statistically significant when p < 0.05.

## Supplementary information

Supplementary Information

## Data Availability

The datasets generated during and/or analysed during the current study are available from the corresponding author on reasonable request.
